# The role of lateral wall reconstruction in improving surgical outcomes for intertrochanteric femur fractures

**DOI:** 10.1002/jeo2.70385

**Published:** 2025-07-24

**Authors:** Sanaz Bordbar, Mehdi Komijani, Fereshteh Eidy, Mohammad Hossein Nabian, Leila Zanjani

**Affiliations:** ^1^ Students' Scientific Research Center Tehran University of Medical Sciences Tehran Iran; ^2^ Center for Orthopedic Transdisciplinary Applied Research (COTAR) Tehran University of Medical Sciences Tehran Iran; ^3^ Department of Orthopedics, School of Medicine Tehran University of Medical Sciences Tehran Iran

**Keywords:** cephalomedullary nail, intertrochanteric fracture, lateral wall, PFNA, reconstruction

## Abstract

**Purpose:**

Intertrochanteric fractures are the most common type of proximal femur fractures, with lateral wall fractures occurring in approximately 33% of cases. This meta‐analysis compared the effectiveness of intramedullary fixation methods, specifically the proximal femoral nail (PFN) and the proximal femoral nail anti‐rotation (PFNA), to other treatment options.

**Methods:**

A systematic review was conducted using PubMed, Scopus, Web of Science and Embase, following PRISMA guidelines. We included clinical trials on intertrochanteric femur fractures with lateral wall involvement. Random effects models were used to analyze mean differences across treatment methods, with statistical evaluations including *I*², Cochran's Q, sensitivity analyses, and Egger's test. Additionally, a subgroup analysis was performed.

**Results:**

Of the initial 1009 results, 10 studies involving 516 patients were included. The surgical methods assessed included PFN/PFNA, PFN with trochanteric buttress plates, screw‐augmented PFN, PFNA with a sliding compression plate and InterTan. Blood loss was significantly greater in the experimental models (MD: 31.83, 95% confidence interval [CI]: 0.28–63.38, *p* < 0.001, *I*² = 99%). Union time was reduced in the experimental models (MD: −0.60, 95% CI: −0.95 to −0.24, *p* = 0.18, *I*² = 42%). The Harris hip score (HHS) was also higher (MD: 7.03, 95% CI: 4.81–9.26, *p* = 0.74, *I*² = 0%).

**Conclusion:**

Combining PFN with lateral wall reconstruction techniques may increase blood loss, decrease union times, and improve functional outcomes, suggesting advantages over PFN or PFNA alone.

**Trial Registration:**

The PROSPERO registration number: CRD42024602939.

**Level of Evidence:**

Level II, systematic review of Level II studies.

**Abbreviations**
aPFN: screw‐augmented PFN
BMI: body mass index
HHS: Harris hip scores
IMN: intramedullary fixation
PFN: proximal femoral nail
PFNA: proximal femoral nail antirotation
RCT: randomised control trial
TBP: trochanteric buttress plates

## INTRODUCTION

Intertrochanteric fractures are common among elderly individuals, especially in people with pre‐existing health conditions. They represent the most frequent type of proximal femur fracture. These fractures are particularly prevalent in females and individuals with osteoporosis. They are usually trauma‐related, with falls being the leading cause [8]. The incidence of intertrochanteric fractures is approximately 171 in 100,000 [21].

The lateral wall refers to the cortical bone located lateral to the femoral shaft [5]. The lateral wall is key to the stabilisation of fractures caused by the trochanteric region [3]. It functions as a lateral buttress for the proximal femur fragment [11]. The lateral wall occurs in approximately 33% of intertrochanteric fractures [14]. A lack of stabilisation of the lateral wall in these fractures can lead to postoperative complications, such as subtrochanteric fractures and severe health issues [13]. Factors such as the fixation method, implant choice, and insertion technique affect the stability of the fixation [4].

The primary treatment for intertrochanteric fractures involving the lateral wall is intramedullary fixation (IMN) using a proximal femoral nail (PFN). It prevents medialization of the femur shaft and stabilises the proximal femur fragments. The fixation failure rate of the IMN in cases with lateral wall fractures is approximately 24% [26]. Alternative techniques have been proposed for IMN. Intramedullary nailing combined with locking plates, trochanteric stabilising plates or screw augmentation has also been utilised [22].

Some studies suggest that PFN provides sufficient stabilisation and offers advantages such as shorter operative time and fewer complications [9]. Others have proposed that there is a need for additional stabilisation and reconstruction of the lateral wall [7, 28]. This systematic meta‐analysis aims to evaluate whether IMN alone is sufficient for managing lateral wall fractures or if adjunctive techniques are necessary for optimal stabilisation.

## METHODS

### Bibliographical databases and search strategy

This systematic review was conducted in accordance with the Preferred Reporting Items for Systematic Reviews and Meta‐Analyses (PRISMA) flowchart. Four databases, including PubMed, Scopus, Embase and Web of Science, were systematically searched on 27 June 2024. The research question was developed using the patient, intervention, comparison, and outcome (PICO) model.

Population: Intertrochanteric femur fractures with lateral wall fractures.

Intervention: PFNA, PFN + buttressing by trochanteric buttress plate (TBP), screw‐augmented PFN, PFNA + slidding compression, IMN + wire or screw and IMN + reconstruction plate.

Comparison: intertrochanteric femur fractures without lateral wall fractures.

Outcome: Peri‐implant fracture rate, reoperation rates, duration of surgery, intraoperative blood loss, required blood transfusion, length of hospital stay, functional outcome, mortality rate, Harris hip scores and complications (infection, cutting out, loss of reduction, backing out of lag screws, screw breakage, nail breakage, nonunion and delayed healing rates and femoral head necrosis).

The search strategy was constructed by combining relevant keywords related to the population. The following keywords were included: intertrochanteric fracture, pertrochanteric fracture, lateral wall fracture, lateral wall involvement and their synonym. The detailed search strategy for each database is provided in Supporting Information: Material S1.

### Eligibility criteria

The inclusion criteria were studies investigating treatments for intertrochanteric femur fractures involving the lateral wall. Eligible study designs included cohort studies, cross‐sectional studies, and randomised clinical trials (RCTs). The exclusion criteria were as follows: in vitro studies, biomechanical studies, in vivo studies, conference abstracts, editorials, case reports, case series or reviews, as they do not provide sufficient depth or relevance for our analysis. Additionally, studies in languages other than English were excluded from the analysis.

### Study selection

All retrieved studies were imported into EndNote software, version X9 (Clarivate Plc). First, the duplicates were removed. Two researchers (S.B. and F.E.) independently screened the titles and abstracts. Then, full‐text articles were assessed based on the eligibility criteria. Discrepancies at any stage were resolved by a third researcher (M.K.).

### Quality analysis

The Joanna Briggs Institute (JBI) Critical Appraisal Tool was used to evaluate the methodological quality and risk of bias in the included studies. The JBI framework provides structured criteria for assessing aspects such as study design, sample selection, data collection methods, and statistical analysis [18]. Two researchers independently assessed the risk of bias, and disagreements were resolved through discussion with a third reviewer (M.K.).

### Data extraction

Data were independently extracted by two researchers (S.B. and F.E.), with a third researcher (M.K.) resolving any discrepancies. The following data were extracted: (1) Article‐related information: author, year; (2) intervention‐related factors, such as type, interval between fracture and surgery, duration of surgery, blood loss, quality of reduction, union time, Harris Hip Score (HHS), and complications such as limb shortening, pain, screw penetration, impingement, revision surgery, fixation fracture, implement breakage, nonunion, infection and necrosis; (3) the patients were as follows: age, number, sex and BMI; and (4) fracture‐related factors: cause of the fracture.

### Quantitative analysis

Statistical analysis was performed via the R Meta package, version 4.3.1. A random effect model with mean differences was used, employing mean differences to compare treatment modalities, with PFN or PFNA selected as the control groups. Heterogeneity was assessed using the *I*² statistic and Cochran's Q test. Additionally, sensitivity analyses and subgroup analyses were conducted to assess heterogeneity. Publication bias was evaluated using Egger's test.

## RESULTS

### Study selection

**Figure 1:**
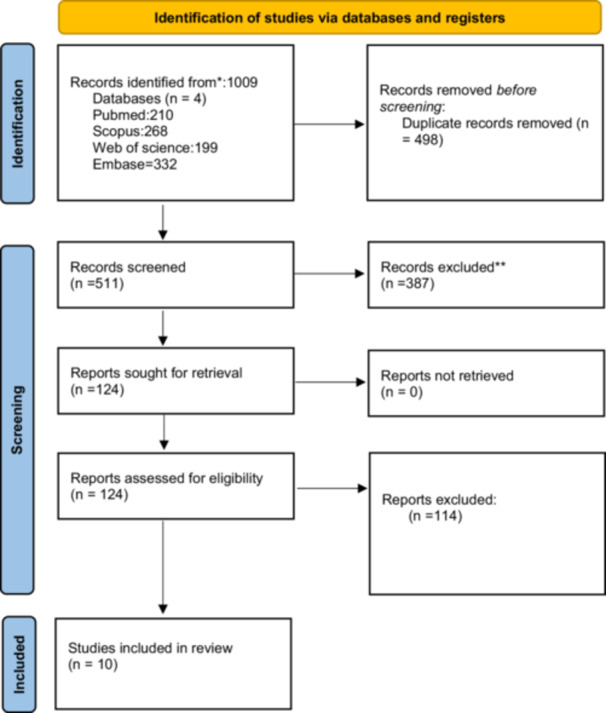
PRISMA flowchart.

The search yielded 1009 results. After duplicate removal, 511 articles remained. Following title and abstract screening, 124 articles were eligible. Following the full‐text screening, 10 articles met the inclusion criteria. Details of the screening process are presented in the PRISMA flowchart (Figure 1).

### Quality analysis

The studies were divided into three groups based on their design, and a separate quality assessment was conducted for each: cross‐sectional studies (Table 1), cohort studies (Table 2) and RCTs (Table 3).

**Table 1:** Quality analysis of the cross‐sectional studies.

Article	Inclusion criteria	Study subjects and setting	Measured exposure	Condition measurement	Confounding factors	Strategies for confounding factors	Measurement of outcomes	Statical analysis
Ganjale et al. [1]	No	Yes	Yes	Yes	No	No	Yes	No
Kim et al. [9]	Yes	Yes	Yes	Yes	No	No	Yes	Yes

**Table 2:** Quality analysis of the cohort studies.

Article	Recruitment of people	Similar measurement	Measured exposure	Confounding factors	Strategies for confounding factors	Groups free of outcome	Measurement of outcomes	Statical analysis
Kulkarni et al. [10]	Yes	Yes	Yes	No	No	Not applicable	Yes	NO
Tang et al. [24]	Yes	Yes	Yes	No	No	Not applicable	Yes	Yes
Rajput et al. [20]	Yes	Yes	Yes	No	No	Not applicable	Yes	Yes
Gao et al. [2]	No	No	No	No	No	Not applicable	Yes	Yes
Jain et al. [6]	Yes	Yes	Yes	No	No	Not applicable	Yes	Yes
Zhang et al. [28]	Yes	Yes	Yes	No	No	Not applicable	Yes	Yes
Wang et al. [27]	Yes	Yes	Yes	No	No	Not applicable	Yes	Yes

**Table 3:** Quality analysis of the RCT studies.

Article	Randomisation	Allocation concealment	Similar groups	Participant blinding	Delivering person concealment	Identical groups	Blinding of outcome assessors	Similar outcome measurement	Reliable measurement	Complete follow‐up	Analyzed in the group	Statical analysis	Appropriate design
Jain et al. [6]	Yes	Unclear	Unclear	Unclear	Unclear	Yes	Yes	Yes	Yes	Yes	Yes	Yes	Yes

Abbreviation: RCT, randomised clinical trial.

### Study characteristics

Overall, 516 patients were included. The surgical methods used were as follows: PFN or PFNA; PFN with TBP; screw‐augmented PFN (aPFN); PFNA with a sliding compression plate; InterTan; PFN with wire or screw; and PFN with a reconstruction plate. The smallest cohort, as reported by Jain et al. [6], consisted of 30 patients, while the largest cohort, as reported by Kulkarni et al. [10], comprised 154 patients. The mean age across studies ranged from approximately 55.3 to 78.08 years. The male prevalence varied from 21% to 87% across studies. This diversity suggests variation in study design, with some potentially focusing on gender‐specific outcomes, while others included more balanced demographics. The average BMI reported in several studies ranged from 21.22 to 23, indicating that participants were generally within a normal to slightly overweight range. Among the six studies that reported fracture laterality, four found that femoral neck fractures were more commonly left‐sided. This finding suggests a possible predominance of left‐sided injuries. The detailed data are presented in Table 4.

**Table 4:** Demographic data, including the number of patients, age, sex, BMI, and side of fracture, were collected.

Author	Year	Type of intervention	Number	Age, years (mean)	Male numbers	BMI kg/m^2^ (mean)	Left fracture numbers
Jain et al. [6]	2022	PFN	30	60.13	26		18
Jain et al. [6]	2022	PFN + TBP	30	60.3	22		14
Tang et al. [24]	2023	PFNA	72	78.07	27	21.22	38
Rajput et al. [20]	2022	aPFN	30	59.03			15
Rajput et al. [20]	2022	PFN	30	60.67			17
Jain et al. [6]	2022	TBP	30	60.3	22		14
Zhang et al. [28]	2023	PFNA + slidding compression	34	73.18	16	22.5	19
Zhang et al. [28]	2023	InterTAN	42	70.95	20	22.8	20
Kulkarni et al. [10]	2017	PFN + wire or screw	74	74			
Kulkarni et al. [10]	2017	PFN	74	74			
Wang et al. [27]	2021	PFN + reconstruction plate	16	74.3	5	22.1	9
Wang et al. [27]	2021	PFN	19	71.3	4	23	9
Kim et al. [9]	2015	PFNA	44	66	21		
Gao et al. [2]	2017	Many methods	99	70.4	42		
Ganjale et al. [1]	2018	TBP	32	55.3	18		

Abbreviations: BMI, body mass index; PFN, proximal femoral nail; PFNA, the proximal femoral nail anti‐rotation; TBP, trochanteric buttress plate.

### Causes of fracture

The causes of intertrochanteric fractures varied among the groups treated with different surgical interventions. Among the 268 reported cases, 51.5% were due to road traffic accidents, 29.9% to falls, and 16% to slipping. Falling was the most common cause among patients treated with PFN, while road traffic accidents were more prevalent in the TBP group, slipping in the aPFN group, and road traffic accidents in the sliding compression plates group. The detailed data are presented in Table 5.

**Table 5:** Fractures can lead to the need for intertrochanteric surgeries for lateral wall fractures.

Author	Type of intervention	Road traffic accident	Falling	Slip on the ground	Other reasons
Jain et al. [6]	PFN	22	8		0
Jain et al. [6]	PFN + (TBP)	22	6		2
Tang et al. [24]	PFNA				
Rajput et al. [20]	aPFN	6	3	21	
Rajput et al. [20]	PFN	5	5	20	
Jain et al. [6]	PFN + TBP	22	6	0	2
Zhang et al. [28]	PFNA + sliding compression plate	20	14		
Zhang et al. [28]	InterTAN	23	19		
Kulkarni et al. [10]	PFN + wire or screw				
Kulkarni et al. [10]	PFN				
Wang et al. [27]	PFN + reconstruction plate	2	13		1
Wang et al. [27]	PFN	1	16		2
Kim et al. [9]	PFNA				
Gao et al. [2]	Many methods	15	3		2
Ganjale et al. [1]	TBP				

Abbreviations: PFN, proximal femoral nail; PFNA, the proximal femoral nail anti‐rotation; TBP, trochanteric buttress plate.

### Surgery‐associated factors

The follow‐up time ranged from as short as 12 weeks to as long as 4.16 years, indicating variability in study duration. The surgical time ranged from 35.2 to 101.62 minutes, with PFN combined with a wire or screw having the shortest mean time, and PFNA with a sliding compression plate having the longest. Blood loss ranged from as low as 93 mL to as high as 228.4 mL, indicating differing levels of intraoperative haemorrhage depending on the surgical technique employed. The greatest amount of blood loss was associated with the PFN with a reconstruction plate, and the lowest amount was associated with the aPFN. The union time generally ranged from approximately 3.61 months to 14.2 months, with a notable difference, particularly between the PFNA technique and other methods. The longest union time was observed in the aPFN group, and the shortest union time was reported for PFN combined with a wire or screw. Detailed data are presented in Table 6.

**Table 6:** Surgery‐related outcomes: Duration, blood loss and time to union.

Author	Type of intervention	Follow‐up time, weeks (mean)	Duration of surgery, minutes (mean)	Duration of surgery, minutes (SD)	Blood loss, mL (mean)	Blood loss, mL (SD)	Union time, weeks (mean)	Union time, weeks (SD)
Jain et al. [6]	PFN	16.2	64.88	12.24	93	1.18	13.4	
Jain et al. [6]	PFN+ (TBP)	16.2	91.86	12.78	144.8	3.6	11.6	
Tang et al. [24]	PFNA	4.16 years	54.1	8.74	228.4	48.8	3.96	0.7
Rajput et al. [20]	aPFN		68.67	8.99	104	4.32	12.66	1.68
Rajput et al. [20]	PFN		67.17	5.82	103	13.17	13.47	1.47
Jain et al. [6]	PFN+ TBP		91.9	12.8	144.8	3.6	11.6	
Zhang et al. [28]	PFNA+ sliding compression plate	15.12	101.62	16.73	123.24	18.38		
Zhang et al. [28]	InterTAN	13.69	89.29	12.57	112.14	21.81		
Kulkarni et al. [10]	PFN + wire or screw		44	3.17			3.61	0.746
Kulkarni et al. [10]	PFN		35.2	3.5			4.1	0.764
Wang et al. [27]	PFN + reconstruction plate	12.5	91	36.2	226.5	52.5	11.8	3.6
Wang et al. [27]	PFN		63.1	32.2	154.3	48.3	14.2	2.8
Kim et al. [9]	PFNA	12						
Gao et al. [2]	Many methods	39.1						
Ganjale et al. [1]	TBP	10.6	75		180			

Abbreviations: PFN, proximal femoral nail; PFNA, the proximal femoral nail anti‐rotation; TBP, trochanteric buttress plate.

### Quality of reduction and HHS

The quality of reduction was categorised into three groups: good, acceptable, and unacceptable. The combined proportion of good and acceptable reductions, compared to unacceptable ones, was highest for PFN combined with either a reconstruction plate or a sliding compression plate, and lowest for the InterTAN method. Additionally, the HHS was reported in several studies. The combined excellent and good outcomes were highest for PFN with the TBP method, and lowest for PFN without augmentation (Table 7).

**Table 7:** Quality of reduction, and HHS following intertrochanteric surgeries with lateral wall fractures.

Author	Type of intervention	Quality of reduction good	Quality of reduction acceptable	The quality of reduction is unacceptable	HHS excellent	HHS good	HHS fair	HHS poor	HHS_mean‐12 month	HHS_12 month (SD)
Jain et al. [6]	PFN				24			4		
Jain et al. [6]	PFN + (TBP)				29			1		
Tang et al. [24]	PFNA	46	20	6					83.8	6.6
Rajput et al. [20]	aPFN				28			1		
Rajput et al. [20]	PFN				25			5		
Jain et al. [6]	PFN + TBP				25	1	0		94.1	7.5
Zhang et al. [28]	PFNA + slidding compression	23	9	2					91.71	7.37
Zhang et al. [28]	InterTAN	22	14	6					84.33	8.63
Kulkarni et al. [10]	PFN + wire or screw				46		17	14		
Kulkarni et al. [10]	PFN				26		32	19		
Wang et al. [27]	PFN + reconstruction plate	11	4	1	11					
Wang et al. [27]	PFN	10	6	3	10					
Kim et al. [9]	PFNA	21	21	2						
Gao et al. [2]	Many methods									
Ganjale et al. [1]	TBP									

Abbreviations: PFN, proximal femoral nail; PFNA, the proximal femoral nail anti‐rotation; TBP, trochanteric buttress plate.

### Complications

Various postoperative complications are common. Reduction loss was the most common, followed by revision surgery, malunion, implant breakage, screw migration, nonunion, and infection. The overall complication rate ranged from 0% to 57%, depending on the surgical method. The highest complication rates were associated with PFN or PFNA, while the lowest rates were observed with TBP (Table 8).

**Table 8:** Number of complications of intertrochanteric surgeries with lateral wall fractures.

Author	Type of intervention	Screw migration	Reduction loss	Implant breakage	Malunion	Nonunion	Infection	Revision Surgery
Jain et al. [6]	PFN	2				0	0	
Jain et al. [6]	PFN + TBP	0				0	0	
Tang et al. [24]	PFNA		1	1	3	2	0	
Rajput et al. [20]	aPFN			1	1		1	1
Rajput et al. [20]	PFN			1	4		1	5
Jain et al. [6]	PFN + TBP	0				0	0	
Zhang et al. [28]	PFNA + sliding compression plate	2	0	0		0		
Zhang et al. [28]	InterTAN	1	1	1		1		
Kulkarni et al. [10]	PFN + wire or screw				5	0		1
Kulkarni et al. [10]	PFN				9	1		7
Wang et al. [27]	PFN + reconstruction plate		4					
Wang et al. [27]	PFN							
Kim et al. [9]	PFNA		25					
Gao et al. [2]	Many methods			4		2		11
Ganjale et al. [1]	TBP	2					2	

Abbreviations: PFN, proximal femoral nail; PFNA, the proximal femoral nail anti‐rotation; TBP, trochanteric buttress plate.

## QUANTITATIVE ANALYSIS

### Blood loss

Four studies with 243 participants reported blood loss. We found significant differences in blood loss among the different treatment methods (MD: 31.83, 95% CI: 0.28–63.38), with heterogeneity (*p* < 0.01, *I* ^2^ = 99%). Egger's test for bias was not significant (*t* = −1.12, *p* = 0.377). Sensitivity analysis revealed that omitting the study conducted by Rajput (95% CI: [−3.96 to 5.96]) could alter the overall result. Overall, the intervention groups (methods other than PFN) in all studies showed increased blood loss compared to the control groups (PFN). Subgroup analysis revealed a pooled mean difference (MD) in blood loss of 31.83 mL (95% CI: 0.28–63.38, *p* = 0.048), indicating a statistically significant difference between groups.

**Figure 2:**
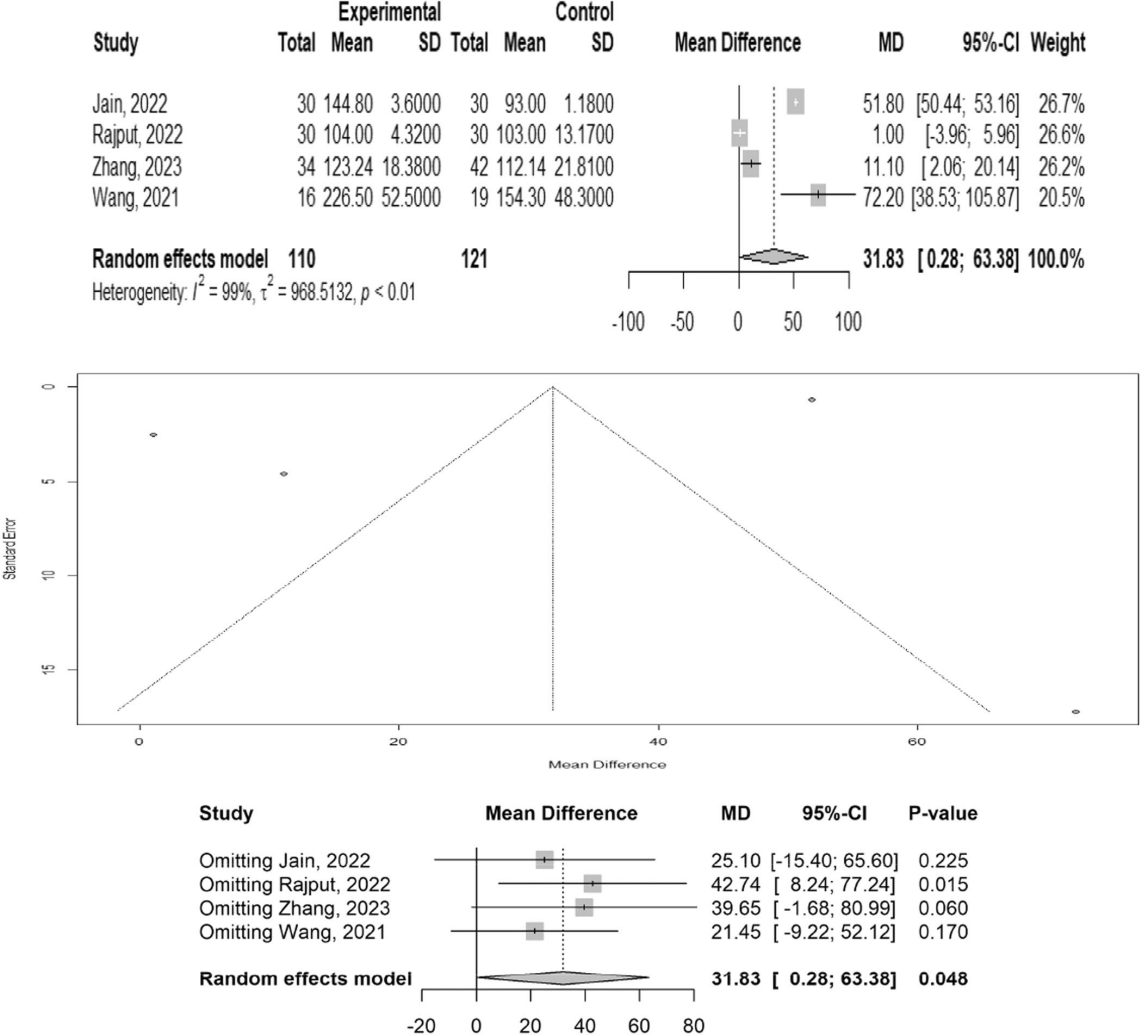
Meta‐analysis of differences in blood loss among different treatment methods. CI, confidence interval; SD, standard deviation.

Zhang et al. [28] and Rajput et al. [20] reported mean differences of 39.65 mL (*p* = 0.060) and 42.74 mL (*p* = 0.015), respectively. Jain et al. [6] and Wang et al. [27] reported modest, non‐significant differences. The variability in effect sizes suggests that blood loss outcomes may be influenced by methodological or clinical factors. Overall, the intervention group experienced significantly greater blood loss, accompanied by substantial between‐study heterogeneity (Figure 2).

### Union time

**Figure 3:**
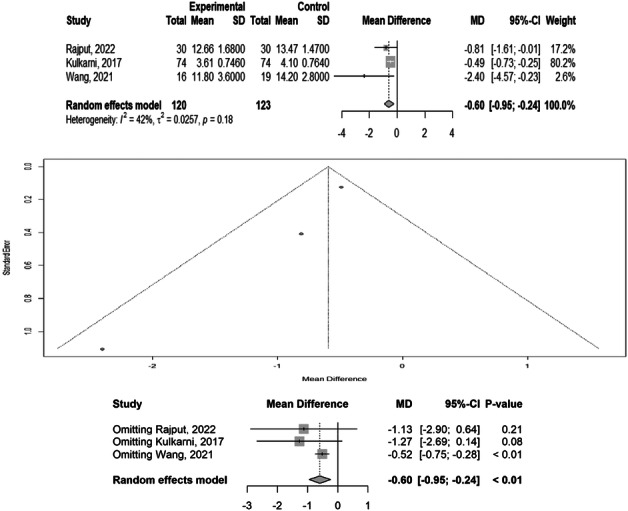
Meta‐analysis of differences in union time among different treatment methods. CI, confidence interval; SD, standard deviation.

Three studies with 243 participants reported union time. We found significant differences in union time across the different treatment methods (MD: −0.60, 95% CI: −0.95 to −0.24), with low heterogeneity (*p* = 0.18, *I*² = 42%). Egger's meta‐bias was not significant (*t* = −4.18, *p* = 0.1530). A sensitivity analysis revealed that omitting the study by Kulkarni et al. [10] (95% CI: [−0.73 to −0.25]) could change the results. Overall, the intervention groups in the studies showed decreased union times compared to the control groups. Subgroup analysis revealed a statistically significant reduction in union time (MD = −0.60, 95% CI: −0.95 to −0.24, *p* < 0.01), favouring the intervention group. Among the individual studies, Wang et al. [27] reported the most substantial and statistically significant effect (MD = −0.52, *p* < 0.01). In contrast, Kulkarni et al. [10] demonstrated a trend toward benefit (MD = −1.27, *p* = 0.08). Overall, the analysis highlights a consistent beneficial effect of the intervention. The observed heterogeneity suggests variability in treatment effects across different populations or methodologies. These findings support the effectiveness of intervention, although the magnitude of benefit may vary across different clinical contexts (Figure 3).

### HHS

**Figure 4:**
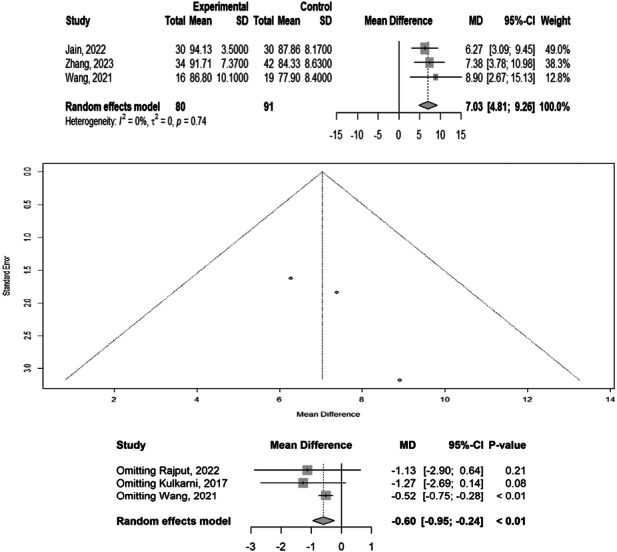
Meta‐analysis of differences in HHS among different treatment methods. CI, confidence interval; HHS, Harris Hip score; SD, standard deviation.

Three studies involving 243 participants reported HHSs. We found significant differences in HHS among the different treatment methods (MD: 7.03, 95% CI: 4.81–9.26), with heterogeneity (*p* = 0.74, *I*² = 0%). Egger's meta‐bias was not significant (*t* = 2.22, *p* = 0.2695). The sensitivity analysis revealed that omitting any of the studies did not affect the HHS. Overall, the HHS was higher in the intervention groups than in the control groups. The subgroup analysis revealed a statistically significant overall effect, with a pooled mean difference of 7.03 (95% CI: 4.81–9.26, *p* < 0.01), indicating a positive impact of the intervention. Wang et al. [27] reported the most substantial and statistically significant effect (MD = 7.52, *p* < 0.01), whereas Kulkarni et al. [10] reported a mean difference of 1.27 (*p* = 0.08). The observed lack of heterogeneity suggests consistent treatment effects across studies, supporting the effectiveness of the intervention; however, the magnitude of benefit may vary across different clinical contexts (Figure 4).

## DISCUSSION

In our study, we included 10 studies that discussed different methods in comparison with the PFN method for treating intertrochanteric fractures with lateral wall fractures. Compared with PFN, other methods are associated with increased blood loss, decreased union time, and increased HHS.

The majority of the patients were elderly, which is consistent with demographic trends in hip fractures. Hip fractures are a leading cause of morbidity and mortality in elderly individuals. As a result, targeted treatment methods are crucial for maximising patient outcomes [25]. Additionally, older people have greater risks of osteoporosis, which can lead to perioperative fractures, an increased risk of periprosthetic fractures, and late aseptic loosening. These factors also influence surgical choice; for example, the choice between cemented and cementless implants is affected by osteoporosis [16, 19]. Restoring the integrity of the lateral wall is essential for preventing further complications such as refracture or malunion [2, 3, 17]. The PFN or PFNA provides stability to the posterior and medial parts of the trochanteric. According to the literature, PFN is linked to favourable clinical results, such as pain relief, better functional recovery, and adequate fracture healing without the need for additional fixation devices. This implies that PFN can offer enough stability to promote healing even in the face of intricate fracture patterns; this finding is especially noteworthy [9, 24]. Other studies have reported that it does not adequately secure the lateral wall and is associated with a high risk of complications such as increased union time and reduction loss. Therefore, the risk of collapse, implant failure, and screw breakage increases with this method. This may include optimising surgical techniques rather than PFN, selecting appropriate implants, and addressing patient‐specific risk factors [20, 23]. PFN has been revised by the addition of specific devices, such as screws, plates, or wires, to improve the stability of the lateral wall, allowing for early weight‐bearing, which can improve recovery [12]. TBPs are associated with early weight bearing, faster healing, better hip function, and improved reduction [15]. TBP increases stability, restores anatomy, and prevents certain complications, such as screw migration [1]. Compared with PFN, TBP can be used to fix the lateral wall better and has excellent to good outcomes after fixation; however, blood loss and surgical time increase. Increased blood loss can have a substantial impact on patient outcomes, potentially resulting in hemodynamic instability. This situation may necessitate blood transfusions and increase the risk of complications, including infection and organ dysfunction. Consequently, this can extend the recovery time [6]. The TBP is fixed to the trochanteric via an antirotatal screw and hip screw, and the plate is passed with the guide of the wires. As a result, exposure is reduced, and the whole operation is performed with little expertise. The plate is long enough to buttress the trochanteric area, and it can be used to split or communicate lateral wall fractures with improved reduction quality. The abutment screws used in this method allow the need for controlled collapse for healing [6, 7].

Screw‐augmented PFN can be used as an alternative to PFN in these fractures. It has better functional outcomes, as indicated by the Wilson and Salavati scores. However, it results in more blood loss. Compared with PFN, it is associated with fewer complications, such as limb shortening and screw breakage. Screw augmentation provides better buttressing of the lateral wall, resulting in improved functional outcomes [20]. Augmentation with a wire or screw can result in fewer complications, and improved functional outcomes. However, it is associated with increased surgery time and minimal blood loss [10]. Sliding compression plates, in addition to PFN, can be associated with a reduced failure rate, better mobility, and greater efficiency than PFN methods. Sliding compression plates can be regarded as an effective method for lateral wall fractures [28]. Compared with PFN, reconstruction plates are associated with increased surgery time and blood loss. However, it has better functional outcomes and a lower complication rate [27].

When evaluating the findings and considering their generalisability, the diversity of studies included in our analysis presents both advantages and challenges. The variation in study designs, ranging from prospective studies and randomised controlled trials to cross‐sectional and retrospective investigations, complicates direct comparisons of results across different research settings. Additionally, there were differences in reported outcomes, including variability in how each study measured and described comparable variables. Patient characteristics such as comorbidities and demographic factors play a crucial role in assessing the broader applicability of the results. This variability may lead to differing treatment effects, which can limit the generalisability of the findings to the broader population. Furthermore, our analysis was limited by the lack of osteoporosis‐specific outcomes in the original studies, as well as inconsistent reporting of the relationships between techniques and fracture patterns. These limitations restricted our ability to perform more detailed subgroup analyses of these clinically relevant factors.

## RECOMMENDATIONS

For clinicians managing intertrochanteric fractures with lateral wall involvement, particularly in elderly or osteoporotic patients, the use of TBP in conjunction with PFN is advised to increase stability, minimise reduction loss, and improve functional outcomes. Compared with standard PFN, screw‐augmented PFN is a viable option associated with fewer complications. In instances requiring anatomical restoration, reconstruction plates may be employed, although this may lead to increased surgical duration and blood loss. Standalone PFN should be avoided in osteoporotic patients because of an elevated risk of implant failure, reduction loss, and screw migration. Instead, sliding compression plates or wire augmentation can be beneficial for improved fixation in comminuted fractures. Surgeons are encouraged to adopt techniques that reduce blood loss and surgical trauma while ensuring appropriate lateral wall reconstruction to mitigate the risk of complications such as malunion or refracture. Early weight‐bearing and rehabilitation should be promoted for stable constructs, and consideration of patient‐specific factors (such as bone quality, fracture pattern) is essential for optimal implant selection and outcomes.

## CONCLUSION

To conclude, our study investigated different treatment methods in addition to PFN for the treatment of intertrochanteric fractures with lateral wall damage. Compared with other methods, PFN generally results in less blood loss, longer union times, and lower HHS; moreover, it is associated with higher complication rates, such as reduction loss and malunion. Although PFN is a standard treatment for intertrochanteric fractures, it does not provide sufficient support for the lateral wall of the hip. As a result, it leads to an increased risk of refracture and complications. Reconstruction of the lateral wall is essential for improved outcomes in intertrochanteric fractures. The PFN method can be revised by adding of screws, plates, or wires to enhance the reconstruction of the lateral wall. The proposed treatments, such as TBP, have shown promising results. Overall, PFN alone is insufficient for this type of fracture, and the use of other treatment methods is necessary.

## AUTHOR CONTRIBUTIONS

*Conceptualisation*: Mehdi Komijani and Mohammad Hossein Nabian. *Methodology*: Fereshteh Eidy and Leila Zanjani. *Screening and data extraction*: Sanaz Bordbar, Mehdi Komijani, and Fereshteh Eidy. *Writing‐original draft*: Sanaz Bordbar and Mehdi Komijani. *Writing‐review and editing*: Sanaz Bordbar and Mehdi Komijani. *Project administration*: Mohammad Hossein Nabian and Mehdi Komijani. All the authors read and approved the final manuscript.

## CONFLICT OF INTEREST STATEMENT

The authors declare no conflicts of interest.

## ETHICS STATEMENT

Please include the name of the institutional review board (IRB) and the approval number. If not applicable, please state so. Tehran University of Medical Sciences, IR.TUMS.SHARIATI.REC.1403.104.

## Supporting information

Supplementary Information

## Data Availability

Data can be accessed upon request from the corresponding author.
