# Supplementary material for: The role of lateral wall reconstruction in improving surgical outcomes for intertrochanteric femur fractures
Source: J Exp Orthop. 2025 Jul 24;12(3):e70385. doi: 10.1002/jeo2.70385 (PMC12287866; doi:10.1002/jeo2.70385)
Supplement: Supplementary file 1 — Supplementary Information [file JEO2-12-e70385-s001.docx]

**PICO:**

**Population:**

intertrochanteric femur fractures with lateral wall fractures

**Intervention:**

PFNA, buttressing by trochanteric buttress plate (TBP) supplemented with proximal femoral nailing, Screw-augmented PFN, trochanter stabilizing plate, RoSA/TSP, proximal femoral totally bionic nail (PFTBN), CMN.

**Comparison:**

intertrochanteric femur fractures without lateral wall fractures

**Outcome:**

peri-implant fracture rate, re-operation rates, duration of surgery, intra-operative blood loss, required blood transfusion, lengths of hospital stay, functional outcome, mortality rate, Harris hip scores (1 year), and complications (infection, cutting out, loss of reduction, backing out of lag screws, cephalic screw breakage, nail breakage, nonunion and delayed healing rates, femoral head necrosis)

**1. Pubmed (NML)**

Date of Search: 27/06/2024

Number of results: 210

((("intertrochanteric" AND "fracture") OR ("pertrochanteral" OR "pertrochanteric") OR "Intertrochanteric fracture*" OR "pertrochanteric fracture*" OR "extracapsular fracture*" OR "intertrochanteric HIP fracture" OR "extracapsular hip fracture*" OR "hip fracture*" OR "Hip fractures" [MeSh] OR "Intertrochanteric femoral fracture*" OR "femoral intertrochanteric fracture" OR "intertrochanteric fracture femur" OR "intertrochanteric fracture of femur" OR "intertrochanteric fracture of the femur" OR "Intertrochanteric femur fracture*" OR "Intertrochanteric hip fracture*" OR "Fractures, Intertrochanteric" OR ((AO OR OTA) AND (31A2.2 OR 31A2.3 OR 31A3.1 OR 31A3.2 OR 31A3.3)) OR((hip OR hips OR trochant* OR pertrochant* OR intertrochant* OR extracapsular*) AND fracture*))) AND ("lateral wall fracture*" OR "lateral wall involvement" OR ( lateral and wall))

**2. Scopus (Elsevier)**

Date of Search: 26/06/2024

Number of results: 268

( ( INDEXTERMS ( ( ( ( "intertrochanteric" AND "fracture" ) OR ( "pertrochanteral" OR "pertrochanteric" ) OR "intertrochanteric fracture*" OR "pertrochanteric fracture*" OR "extracapsular fracture*" OR "intertrochanteric hip fracture" OR "extracapsular hip fracture*" OR "hip fracture*" OR "intertrochanteric femoral fracture*" OR "femoral intertrochanteric fracture" OR "intertrochanteric fracture femur" OR "intertrochanteric fracture of femur" OR "intertrochanteric fracture of the femur" OR "intertrochanteric femur fracture*" OR "intertrochanteric hip fracture*" OR "fractures, intertrochanteric" OR ( ( ao OR ota ) AND ( 31a2.2 OR 31a2.3 OR 31a3.1 OR 31a3.2 OR 31a3.3 ) ) OR ( ( hip OR hips OR trochant* OR pertrochant* OR intertrochant* OR extracapsular* ) AND fracture* ) ) ) ) AND INDEXTERMS ( ( "lateral wall fracture*" OR "lateral wall involvement" OR ( lateral AND wall ) ) ) ) ) OR ( ( TITLE-ABS-KEY ( ( ( ( "intertrochanteric" AND "fracture" ) OR ( "pertrochanteral" OR "pertrochanteric" ) OR "intertrochanteric fracture*" OR "pertrochanteric fracture*" OR "extracapsular fracture*" OR "intertrochanteric hip fracture" OR "extracapsular hip fracture*" OR "hip fracture*" OR "intertrochanteric femoral fracture*" OR "femoral intertrochanteric fracture" OR "intertrochanteric fracture femur" OR "intertrochanteric fracture of femur" OR "intertrochanteric fracture of the femur" OR "intertrochanteric femur fracture*" OR "intertrochanteric hip fracture*" OR "fractures, intertrochanteric" OR ( ( ao OR ota ) AND ( 31a2.2 OR 31a2.3 OR 31a3.1 OR 31a3.2 OR 31a3.3 ) ) OR ( ( hip OR hips OR trochant* OR pertrochant* OR intertrochant* OR extracapsular* ) AND fracture* ) ) ) ) AND TITLE-ABS-KEY ( ( "lateral wall fracture*" OR "lateral wall involvement" OR ( lateral AND wall ) ) ) ) )

**3. Web of Science (Clarivate)**

Date of Search: 27/06/2024

Number of results: 199

**(AB=(((("intertrochanteric" AND "fracture") OR ("pertrochanteric" OR "pertrochanteric") OR "Intertrochanteric fracture*" OR "pertrochanteric fracture*" OR "extracapsular fracture*" OR "intertrochanteric HIP fracture" OR "extracapsular hip fracture*" OR "hip fracture*" OR "Intertrochanteric femoral fracture*" OR "femoral intertrochanteric fracture" OR "intertrochanteric fracture femur" OR "intertrochanteric fracture of femur" OR "intertrochanteric fracture of the femur" OR "Intertrochanteric femur fracture*" OR "Intertrochanteric hip fracture*" OR "Fractures, Intertrochanteric" OR ((AO OR OTA) AND (31A2.2 OR 31A2.3 OR 31A3.1 OR 31A3.2 OR 31A3.3)) OR((hip OR hips OR trochant* OR pertrochant* OR intertrochant* OR extracapsular*) AND fracture*))) )) AND AB=(("lateral wall fracture*" OR "lateral wall involvement" OR ( lateral and wall)))**

129

**(TI=(((("intertrochanteric" AND "fracture") OR ("pertrochanteric" OR "pertrochanteric") OR "Intertrochanteric fracture*" OR "pertrochanteric fracture*" OR "extracapsular fracture*" OR "intertrochanteric HIP fracture" OR "extracapsular hip fracture*" OR "hip fracture*" OR "Intertrochanteric femoral fracture*" OR "femoral intertrochanteric fracture" OR "intertrochanteric fracture femur" OR "intertrochanteric fracture of femur" OR "intertrochanteric fracture of the femur" OR "Intertrochanteric femur fracture*" OR "Intertrochanteric hip fracture*" OR "Fractures, Intertrochanteric" OR ((AO OR OTA) AND (31A2.2 OR 31A2.3 OR 31A3.1 OR 31A3.2 OR 31A3.3)) OR((hip OR hips OR trochant* OR pertrochant* OR intertrochant* OR extracapsular*) AND fracture*))) )) AND TI=(("lateral wall fracture*" OR "lateral wall involvement" OR ( lateral and wall)))**

**51**

**(ALL=(((("intertrochanteric" AND "fracture") OR ("pertrochanteric" OR "pertrochanteric") OR "Intertrochanteric fracture*" OR "pertrochanteric fracture*" OR "extracapsular fracture*" OR "intertrochanteric HIP fracture" OR "extracapsular hip fracture*" OR "hip fracture*" OR "Intertrochanteric femoral fracture*" OR "femoral intertrochanteric fracture" OR "intertrochanteric fracture femur" OR "intertrochanteric fracture of femur" OR "intertrochanteric fracture of the femur" OR "Intertrochanteric femur fracture*" OR "Intertrochanteric hip fracture*" OR "Fractures, Intertrochanteric" OR ((AO OR OTA) AND (31A2.2 OR 31A2.3 OR 31A3.1 OR 31A3.2 OR 31A3.3)) OR((hip OR hips OR trochant* OR pertrochant* OR intertrochant* OR extracapsular*) AND fracture*))) )) AND ALL=(("lateral wall fracture*" OR "lateral wall involvement" OR ( lateral and wall)))**

**Combine= 199**

**4. Embase (Elsevier)**

Date of Search: 27/06/2024

Number of results: 332

('intertrochanteric fracture'/exp OR 'pertrochanteral' OR 'pertrochanteric' OR 'extracapsular fracture'/exp OR 'extracapsular hip fracture'/exp OR 'hip fracture'/exp OR 'intertrochanteric femoral fractures' OR 'femoral intertrochanteric fracture' OR 'intertrochanteric fracture femur' OR 'intertrochanteric fracture of femur' OR 'intertrochanteric fracture of the femur' OR 'intertrochanteric femur fracture'/exp OR 'intertrochanteric hip fracture'/exp OR 'fractures, intertrochanteric' OR (ao AND (31a2.2 OR 31a2.3 OR 31a3.1 OR 31a3.2 OR 31a3.3)) OR (('hip'/exp OR hips OR trochant* OR pertrochant* OR intertrochant* OR extracapsular*) AND 'fracture'/exp)) AND ('lateral wall fracture' OR 'lateral wall involvement' OR (lateral AND 'wall'/exp))

35

('intertrochanteric fracture'/exp OR 'intertrochanteric fracture' OR 'pertrochanteral' OR 'pertrochanteric' OR 'extracapsular fracture'/exp OR 'extracapsular fracture' OR 'extracapsular hip fracture'/exp OR 'extracapsular hip fracture' OR 'hip fracture'/exp OR 'hip fracture' OR 'intertrochanteric femoral fractures' OR 'femoral intertrochanteric fracture' OR 'intertrochanteric fracture femur' OR 'intertrochanteric fracture of femur' OR 'intertrochanteric fracture of the femur' OR 'intertrochanteric femur fracture'/exp OR 'intertrochanteric femur fracture' OR 'intertrochanteric hip fracture'/exp OR 'intertrochanteric hip fracture' OR 'fractures, intertrochanteric' OR (ao AND (31a2.2 OR 31a2.3 OR 31a3.1 OR 31a3.2 OR 31a3.3)) OR (('hip'/exp OR hip OR hips OR trochant* OR pertrochant* OR intertrochant* OR extracapsular*) AND ('fracture'/exp OR fracture))) AND ('lateral wall fracture' OR 'lateral wall involvement' OR (lateral AND ('wall'/exp OR wall))

332

Combine=332
